# IL-7-PD-L1 nano-antibody mediated “zipper” effect augments the tumoricidal activity of tumor-infiltrating lymphocytes

**DOI:** 10.1186/s40164-025-00702-y

**Published:** 2025-08-29

**Authors:** Zhongjie Yu, Zhen Guo, Bin Jiang, Yueshu Zhu, Lin Shao, Xinhua Zhang, Yi Zhao, Di Wu, Aotian Xu

**Affiliations:** 1 R&D, Qingdao Sino‑cell Biomedicine Co., Ltd., 1 Changcheng South Road, Chengyang, Qingdao, 266000 Shandong China; 2 Cancer Center, The First Hospital of Jilin University, 1 Xinmin Street, Changchun, 130021 China

**Keywords:** Tumor-infiltrating lymphocytes, Tumor microenvironment, IL-7, PD-L1 nano-antibody, TIGIT, Cytotoxicity

## Abstract

Cancer represents a pressing global health concern, characterized by a substantial number of unmet clinical needs. Cell therapy has emerged as a promising and efficacious approach for cancer treatment, particularly tumor-infiltrating lymphocytes (TILs), which have demonstrated remarkable improvements in patients’ overall survival rates across various clinical studies. However, the tumor microenvironment exerts a adverse effect on TILs, leading to their rapid exhaustion and functional disorder. Consequently, this impedes their ability to effectively eradicate tumors and thus hinders the achievement of the anticipated therapeutic efficacy. Here, we employed lentiviral vector-mediated genetic engineering to manipulate TILs for the expression of TIGIT shRNA, IL-7-PD-L1 nano-antibody fusion protein, and the ‘molecular switch’ HuEGFRt. The engineered TILs exhibited higher viability, reinforced cell expansion, and reduced reliance on IL-2. The stem-like proportion of engineered TILs is significantly augmented, and their activation level is enhanced when co-cultured with tumor cells. Meanwhile, the engineered TILs exert sustained cytotoxicity after repeated stimulation from tumor cells. The use of Cetuximab has been demonstrated in vitro to induce specific apoptosis of engineered TILs through HuEGFRt, thereby ensuring safety throughout the treatment process. In the mouse tumor model, following infusion of engineered TILs, the tumor volume significantly reduced, once again demonstrating the effectiveness of engineered TILs. The findings of our study demonstrate the exceptional performance of engineered TILs, which undoubtedly holds great promise for the clinical application of engineered TILs, ultimately benefiting a larger population of cancer patients.

**Supplementary Information:**

The online version contains supplementary material available at 10.1186/s40164-025-00702-y.

**To the editor**

The tumor-infiltrating lymphocytes (TILs) therapy represents an innovative and promising form of cellular immunotherapy for solid tumors, distinguished by its high safety profile, superior homing capability, and multi-target recognition [1]. However, the clinical efficacy of TILs therapy is challenged by the tumor microenvironment (TME).

In this research, we addressed the therapeutic challenges posed by TME through developing engineered TILs. Through engineered modification, TILs expressing TIGIT shRNA can effectively reduce the expression level of the TIGIT protein, thereby alleviating their functional exhaustion within the TME. Concurrently, these TILs are also equipped with a fusion protein consisting of IL-7 and a PD-L1 nano-antibody. On one hand, the PD-L1 nano-antibody enables specific binding to PD-L1 on tumor cells, thereby blocking the immunosuppressive effects mediated by the PD-L1/PD-1 signaling pathway. On the other hand, the IL-7 component binds to the IL-7 receptor on TILs, enhancing their functional activity. (Fig. 1A, Supplementary Fig. 1A-F) IL-2 plays an essential role in sustaining the survival and proliferation of TILs. However, administering high doses of IL-2 may lead to adverse effects [2]. Our research aims to address TME challenges while simultaneously reduce TILs’ reliance on high-dose IL-2. As depicted in Fig. 1B and Supplementary Fig. 1G-I, the engineered 1# and 3# TILs, expressing the IL-7-PD-L1 nano-antibody fusion protein, which can significantly decrease the reliance on high-dose IL-2. More detailed analysis demonstrated a notable increase in the proportion of stem-like cells (CD39^−^CD69^−^) [3] within both CD8^+^ and CD4^+^ TILs in groups 1# and 3# (Fig. 1C D, Supplementary Fig. 1J K). These findings are consistent with previous studies on peripheral blood T cells [4, 5]. Moreover, in a co-culture system comprising engineered TILs, cancer cells, and dendritic cells, the IL-7-PD-L1 nano-antibody fusion protein may enhance CD80-CD28 interaction by inhibiting the binding of PD-L1 to CD80, thereby promoting the activation of TILs (Fig. 1E, Supplementary Fig. 1L).

**Fig. 1:**
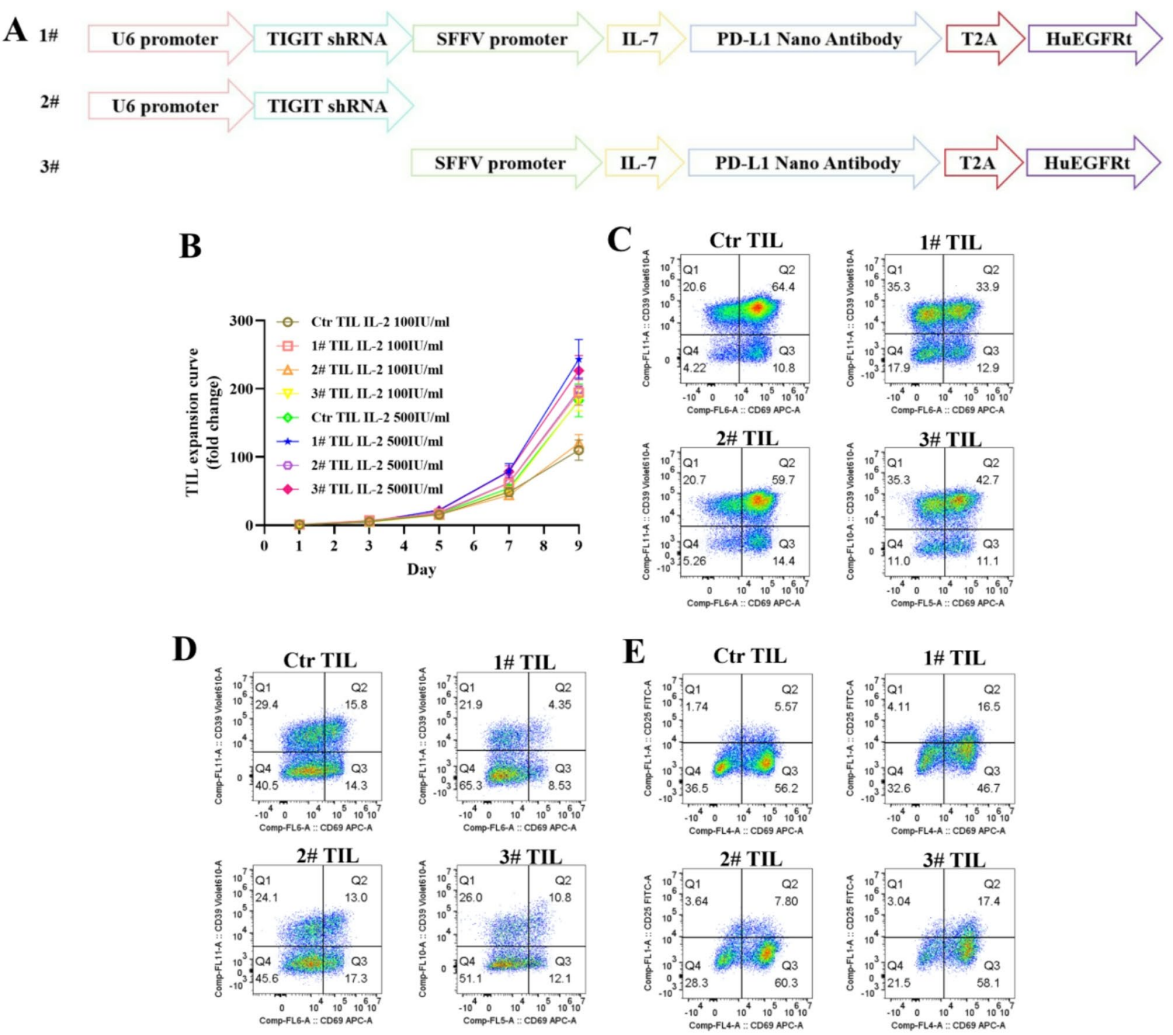
Superior attributes of engineered TILs. (**A**) The molecular framework of engineered TILs. (**B**) The proliferation profiles of engineered TILs cultured in varying concentrations of IL-2. (**C**) The proportion of stem-like cells within the CD8 + subset in engineered TILs cultured under conditions of 100 U/ml IL-2. (**D**) The proportion of stem-like cells within the CD4 + subset in engineered TILs cultured under conditions of 100 U/ml IL-2. (**E**) The proportion of activation in engineered TILs cocultured with cancer cells and dendritic cells

Next, we evaluated the cytotoxic efficacy of engineered TILs against tumor cells. Following exposure to three challenges of tumor cells, we observed that 1# and 3# TILs nearly completely eradicated tumor cells following three rounds of tumor cell challenge (Fig. 2A). The CCK-8 and LDH results provided further evidence that the sustained cytotoxic efficacy of 1# and 3# TILs were markedly superior to other groups (Fig. 2B, Supplementary Fig. 2A). After three consecutive rounds of challenge by tumor cells, the cytotoxicity of TILs against tumor cells declined. This reduction may be attributed to tumor cell-induced exhaustion and dysfunction of TILs. However, in the 1# and 3# TIL groups, the decline in cytotoxicity was less pronounced compared to the Ctr and 2# TIL groups. This suggests that the fusion protein expressed by engineered TILs may, to some extent, inhibit tumor cell-mediated TIL exhaustion and dysfunction (Fig. 2B). Furthermore, the secretion levels of IFN-γ and Granzyme B in 1# and 3# TILs were significantly higher than other groups. (Supplementary Fig. 2B C). These findings reinforce the conclusion that 1# and 3# TILs possess enhanced cytotoxic activity. To comprehensively investigate the role of the IL-7-PD-L1 fusion protein in enhancing TILs-mediated tumor cell killing, we developed a novel engineered 4#TILs (Supplementary Fig. 3A), which is capable of independently expressing IL-7 and PD-L1 nanobodies. Under the equivalent transduction efficiency (Supplementary Fig. 3B), the results of repeated killing assay revealed that the cytotoxic effects of 4# TILs were less significant than those of 1# TILs (Supplementary Fig. 3C). Other experimental results, like CCK-8, LDH, IFN-γ and Granzyme B detection, further confirm that the cytotoxicity of #1 TILs is significantly greater than that of #4 TILs (Supplementary Fig. 3D-G).

**Fig. 2:**
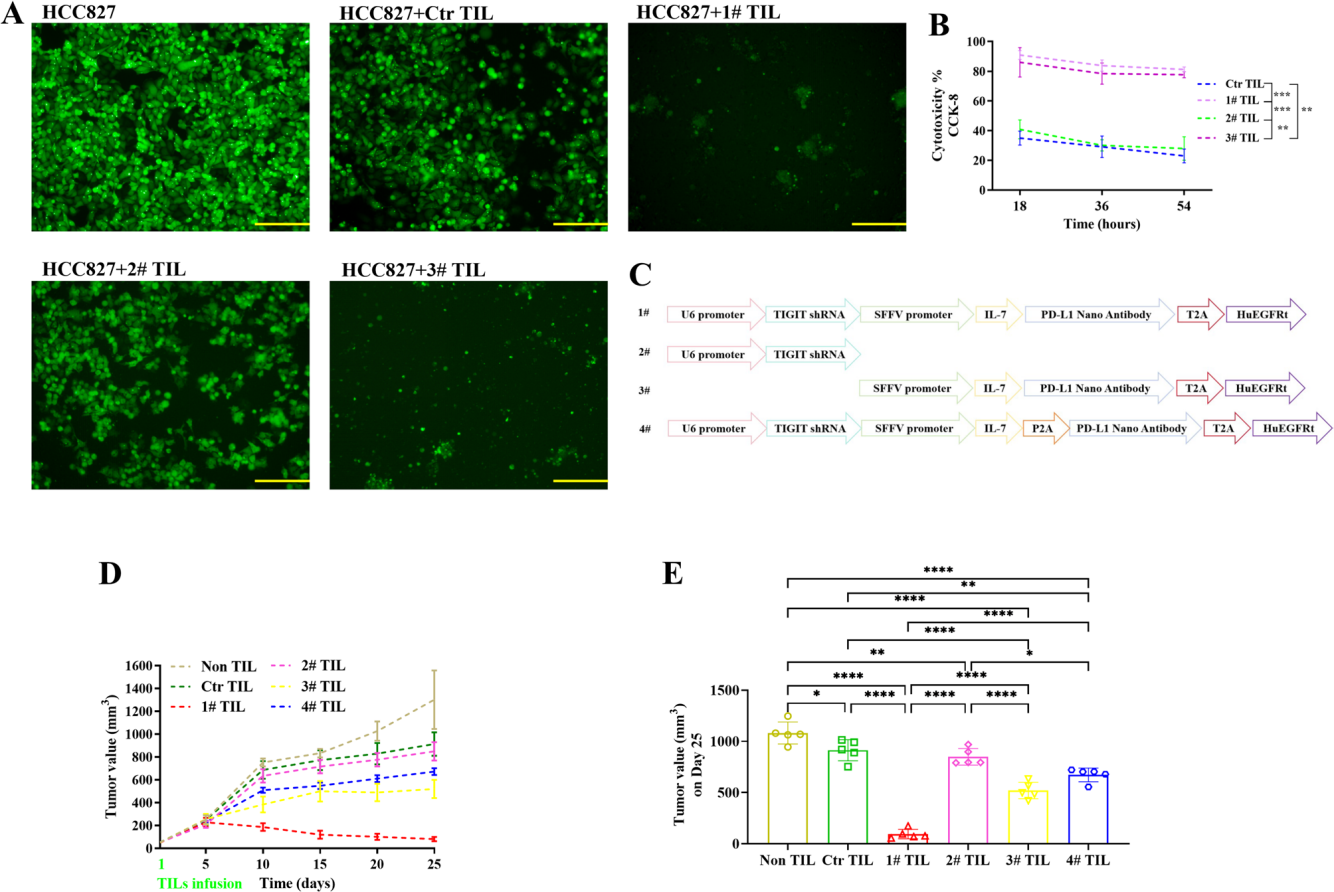
Tumoricidal activity of engineered TILs. (**A**) Following three rounds of challenge of the engineered TILs with tumor cells (GFP-labeled), the efficacy of tumor cell clearance was observed and photoed using fluorescence microscopy. bar = 100 μm (*n* = 3). (**B**) Following sustained challenge of the engineered TILs with tumor cells, the efficacy of tumor cell clearance was quantified by CCK-8 assay (*n* = 3). (**C**) The molecular framework of engineered TILs. (**D**) Engineered TILs shown remarkable tumor-clearance capabilities in vivo (*n* = 5). (**E**) Statistical analysis of the in vivo tumor-clearance capabilities of engineered TILs (*n* = 5). *p < 0.05, **p < 0.01, ***p < 0.001, and ****p < 0.0001

In addition, we established a murine CDX model to evaluate the in vivo tumor clearance efficacy of engineered TILs (Fig. 2C). As shown in Fig. 2D, we systematically monitored tumor volume changes. On day 10, the tumor volume in the 1# TIL group began to decrease, and differences among experimental groups became apparent. As time progressed, these differences increased. On day 25, following ethical guidelines, we ended the experiment and analyzed the tumor volumes across all groups statistically. In vivo findings showed that 1# TILs had the strongest tumor clearance capability, and compared to 3# TILs, the in vivo efficacy of 1# TILs were significantly higher, likely due to TIGIT shRNA effectively inhibiting TIGIT-mediated T cell exhaustion (Fig. 2E) [6].

These data revealed that the fusion expression of IL-7 and PD-L1 nano-antibody represents a critical factor in augmenting the anti-tumor activity of TILs. This bifunctional protein simultaneously binds to the IL-7 receptor on TILs and PD-L1 on tumor cells, functioning as a “zipper” that reduces the spatial separation between TILs and tumor cells, thereby enhancing the tumoricidal activity of TILs.

Safety concerns are a top priority in T-cell therapy, particularly in gene-edited T-cell therapies [7]. In this study, we incorporated the “molecular switch” HuEGFRt into engineered TILs. Upon cetuximab binding, this “molecular switch” specifically triggers apoptosis of engineered TILs (Supplementary Fig. 4A-D) via CDC [8] and ADCC [9] mechanisms. Besides that, throughout the entire in vivo experimental period, no significant changes in body weight were observed in the mice of the engineered TILs group (Supplementary Fig. 4E). Meanwhile, no abnormalities were found in the blood routine and blood biochemical tests. (Supplementary Table 1, Supplementary Table 2). These findings substantiate the safety profile of engineered TILs.

To date, this study has demonstrated for the first time that engineering TILs to express a fusion protein comprising IL-7 and a PD-L1 nano-antibody significantly enhances the tumor-clearing capability of TILs. The engineered TILs exhibited robust therapeutic efficacy while maintaining an excellent safety profile. We anticipate that this novel type of engineered TILs will exhibit faster symptom relief with a lower cell dose, particularly showing improved efficacy in patients with positive PD-L1 expression. The design of our engineered TILs is broadly applicable and can be extended to the treatment of multiple cancer types, offering hope to a greater number of advanced tumor patients.

## Supplementary Information

Below is the link to the electronic supplementary material.

Supplementary Material 1

## Data Availability

No datasets were generated or analysed during the current study.
