# Supplementary material for: IL-7-PD-L1 nano-antibody mediated “zipper” effect augments the tumoricidal activity of tumor-infiltrating lymphocytes
Source: Exp Hematol Oncol. 2025 Aug 29;14:112. doi: 10.1186/s40164-025-00702-y (PMC12398053; doi:10.1186/s40164-025-00702-y)
Supplement: Supplementary file 1 — Supplementary Material 1 [file 40164_2025_702_MOESM1_ESM.docx]

**Supplemental data**

**IL-7-PD-L1 nano-antibody mediated** **"zipper" effect** **augments the tumoricidal activity of tumor-infiltrating lymphocytes**

Zhongjie Yu ^1^, Zhen Guo ^2^, Bin Jiang ^1^, Yueshu Zhu ^1^, Lin Shao ^1^, Xinhua Zhang ^1^, Yi Zhao ^1^, Di Wu ^2#^, Aotian Xu ^1#^

^1^ R&D, Qingdao Sino‑cell Biomedicine Co., Ltd., Qingdao, 266000, China.

^2^ Cancer Center, The First Hospital of Jilin University, Changchun, 130021, China.

#Correspondence to:

Dr Di Wu, Cancer Center, The First Hospital of Jilin University, 1 Xinmin Street, Changchun, 130021, China. E‑mail: wudi1971@jlu.edu.cn.

Dr Aotian Xu, Qingdao Sino‑cell Biomedicine Co., Ltd., 1 Changcheng South Road, Chengyang, Qingdao, Shandong, 266000, China.

E‑mail:xuaotian@sino-cellbiomed.com

**Material and methods**

**Cell culture**

293T and HCC827 cells were also cultured in DMEM and RPMI-1640, respectively. (BDBio, Hangzhou, China) containing 10% Fetal Bovine Serum (BDBio, Hangzhou, China) and 1% penicillin/streptomycin (Solarbio, Beijing, China). All cells were incubated at 37 C with 5% CO_2_.

**Generation of TIL**

Tissue samples were obtained from patients with lung cancer at the Cancer Center of the First Hospital of Jilin University (Approval Number: AF-IRB-026-01). A total of three tissue samples were obtained from three individual patients for the isolation and preparation of TILs. Each replicate in the experiments was derived from TILs isolated from a distinct patient. TIL isolation was performed as previously described with some modifications. ^1^ The tumor was sliced with a scalpel into small pieces, about 1mm^3^ in size and placed in culture in 24-well plates in 2 ml complete medium (CM) containing 10% Human AB serum (GemCell™, Woodland, USA), 1% GlutaMax (Gibco™, New York, USA), 1% penicillin/streptomycin (Solarbio, Beijing, China) and 6,000 IU/ml IL-2 (Sihuan Biotech, Beijing, China) in Advanced RPMI 1640 medium (Gibco™, Paisley, UK). Half media changes were performed every 3 days, to maintain a cell concentration in the range of 0.5~1.0E6 cells/ml. TILs were harvested after removing tumor tissue within 15 days.

**Lentivirus infection and rapid expansion**

Plasmid 1#, 2#, 3# ,4# were constructed and used for lentivirus (LV) packaging (Hillgene, Suzhou, China). The infection of 5E6 TILs was achieved by LV at multiplicity of infection=20 in 1 ml rapid expansion protocol complete medium (REP-CM) containing 50% Advanced RPMI 1640, 50% AIM-V medium (Gibco™, Waltham, USA), and final concentration at 10% Human AB serum, 1% GlutaMax and 1,000 IU/ml IL-2. After 24 hours, the medium containing LV was removed, and TILs were subsequently cultured in fresh REP-CM. After infecting TILs with lentivirus for 48 hours, the cells were collected and assessed the infection efficiency by flow cytometry, based on the percentage of EGFR+ TILs. Meanwhile, use uninfected normal TILs as a control group.

For TILs rapid expansion, 2E5/groups were co-incubated with 2E6 irradiated K562-mIL21-4-1BBL cells in 24-well high-density cell culture plate (GC-BIO. TEC, Shanghai, China) with 8ml REP-CM. The medium was half-changed every two days. TILs were collected for cell counting by using Countstar RigelS2 on day 9.

**Repeated killing assay**

1.5E5 HCC827 cells were seeded in 24-well plate (JET BIOFIL, Guangzhou, China), and then TILs were added into the plate at E:T=5:1. Following an 18-hour interval, the TILs were subsequently restimulated with 1.5E5 HCC827 cells, total three times. Supernatants were collected for subsequent experimental analysis.

**TILs activation**

TILs were co-cultured with HCC827 and dendritic cells (1:2:2) in REP-CM for 24h, and the activation of TILs were assessed using flow cytometry.

**CDC&ADCC assay**

For CDC assay, TILs were cultured in REP-CM containing 25% Baby Rabbit Complement (RUO Cedarlane, CL3441-S-R) and 100 μg/ml Cetuximab (Merk Healthcare KGaA, C10010069). After 24h, cell apoptosis levels were assessed using apoptotic kit (BD BIOSCIENCES PHARMINGEN, 559763).

For ADCC assay, NK cells were isolated from the peripheral blood of healthy volunteers and cultured following the procedures outlined in the kit instructions (CT-002, Stemery). Subsequently, these cells were utilized in the ADCC experiment. TILs were co-cultured with NK cells at E:T=1:10 in REP-CM containing 100 μg/ml Cetuximab. After 24h, cell apoptosis levels were assessed using apoptotic kit

**ELISA assay**

The concentration of IL-7, IFN-γ, and Granzyme B derived from cellular supernatant was detected by Human IL-7 Precoated ELISA Kit (DAKEWE, Shenzhen, China), Human IFN-γ Precoated ELISA Kit (DAKEWE, Shenzhen, China), and Human Granzyme B Precoated ELISA Kit (DAKEWE, Shenzhen, China), respectively. The cellular supernatant was collected following centrifugation, and the levels of secreted IL-7, IFN-γ were quantified using an ELISA assay in accordance with the manufacturer's instructions.

**LDH Cytotoxicity Assay**

The concentration of LDH derived from cellular supernatant was detected by LDH Cytotoxicity Assay Kit (Yeasen, Shanghai, China). The cellular supernatant was collected following centrifugation, and the LDH test assay was performed as the manufacturer's instructions.

**Flow cytometry analysis**

5E5 TILs per group were collected for flow cytometry testing. Cells were first washed in wash buffer (1×PBS (Solarbio, Beijing, China) with 2% bovine serum albumin (TransGen Biotech, Beijing, China). Surface Fc receptors were blocked for 10 min at room temperature using Fc Receptor Blocking Solution (BioLegend, San Diego, USA). Cell surface expression assessment for this study was done using fluorochrome-conjugated antibodies against CD25(FITC, BC96, BioLegend, San Diego, USA), CD69(APC, FN50, BioLegend, San Diego, USA), CD45RA (FITC, HI100, BioLegend, San Diego, USA), CD69L (APC, DREG-56, BioLegend, San Diego, USA), TIGIT (PE, A15153G, BioLegend, San Diego, USA), EGFR (APC, me1B3, Invitrogen, Carlsbad, USA). 7-AAD Viability Staining Solution (BioLegend, San Diego, USA) was used to exclude dead cells from analysis. Samples were then subjected to a flow cytometry analysis (Beckman, Suzhou, China), and the data were analyzed using FlowJo-V10 software.

**Western blotting**

2E6 cells were lysed for 15 min on ice in RIPA lysis buffer (20115ES60, Yeasen), and then treated for 10min at 98℃ in loading buffer (20315ES20, Yeasen). The samples were subjected to 12% SDS-PAGE and transferred to PVDF membranes. The PVDF membranes were blocked in 5% nonfat milk (P0216-300g, Beyotime) at room temperature for 1h with slightly shaking. Blots were probed with primary antibodies IL-7 Monoclonal Antibody (0.50 µg/ml, 500-M07, Thermofisher) at 4 ℃ overnight. After washed with TBST, the membranes were incubated in horseradish peroxidase (HRP)-conjugated secondary antibodies (1:20000, 31430, Invitrogen) at room temperature for 1h. After washed with TBST, enhanced ECL detection reagent (FORSCIENCE) was used to visualize antigen–antibody complexes.

**Mouse model and treatment**

5E6 HCC827 cells/mice were implanted subcutaneously into the right flank of 6-week-old male NSG mice. When the tumor volume reached approximately 50mm^3^, mice were intravenously administered a single dose of 1E7 TILs. Tumor volume and body weight were measured every five days.

**Statistical analysis**

Statistical analyses were determined by one-way ANOVA and two-way ANOVA, followed by Tukey’s multiple comparisons test and Šídák's multiple comparisons test, respectively. All statistical analyses were conducted using GraphPad Prism 9. Significance of differences is reported as *p < 0.05, **p < 0.01, ***p < 0.001, and ****p < 0.0001.

**Acknowledgements**

Thanks are due to Hao Wu, Yurun An, Wenshu Zhu, and Jun Shao for assistance with the experiments.

**Funding**

The present study was supported by the New Industry Cultivation Program of Qingdao (grant no. 23-1-4-xxgg-18-nsh) and the Technological SMEs Innovation Ability Improvement Project of Shandong Province (grant no. 2023TSGC0510).

**Availability of data and material**

The data generated in the present study may be requested from the corresponding author.

**Authors’ contributions**

Project design and administration: Zhongjie Yu, Aotian Xu, Di Wu;

Experiments and data analysis: Zhongjie Yu, Aotian Xu, Zhen Guo, Bin Jiang, Yueshu Zhu, Lin Shao, Xinhua Zhang, Yi Zhao;

Writing-original draft preparation: Zhongjie Yu, Aotian Xu, Zhen Guo.

Writing-review and editing: Di Wu, Aotian Xu.

All authors have read and agreed to the published version of the manuscript.

**Ethics approval and consent to participate**

The clinical sample collection was performed according to the protocols approved by the Ethics Committee of the First Hospital of Jilin University (Changchun, China; Approval Number: AF-IRB-026-01). All patients provided written consented for participation in the present study.

**Patient consent for publication**

The patients provided consent for their information to be published.

**Competing interests**

The authors declare that they have no competing interests.

**Reference**

1 Shah, P. *et al.* Combined IL-2, agonistic CD3 and 4-1BB stimulation preserve clonotype hierarchy in propagated non-small cell lung cancer tumor-infiltrating lymphocytes. *J Immunother Cancer* **10**, doi:10.1136/jitc-2021-003082 (2022).

**Supplementary figure and figure legends**


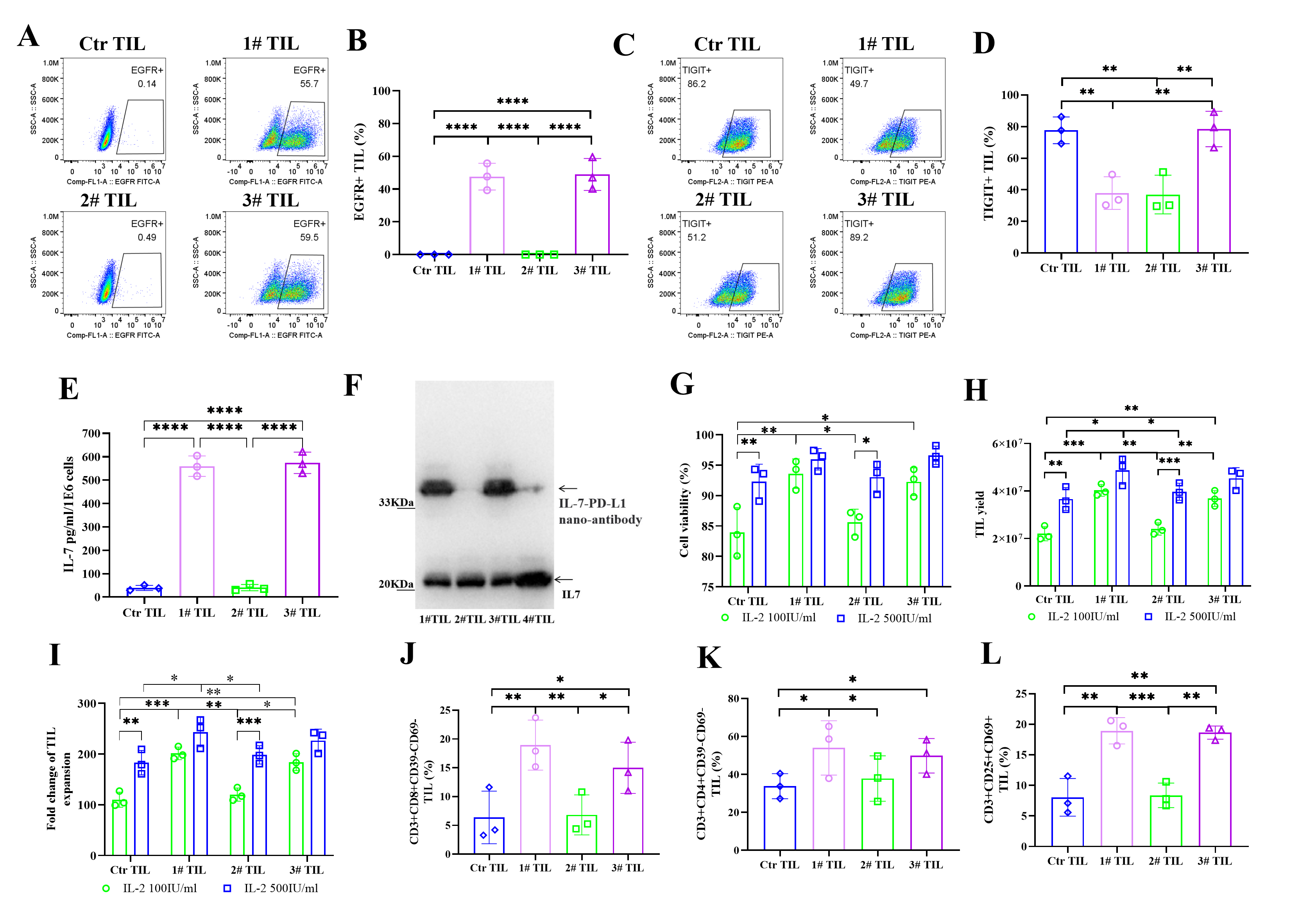


**Supplementary figure 1 Superior attributes of engineered TILs.**

**A** Following the introduction of exogenous genes into TILs via lentiviral vectors, the transduction efficiency (EGFR+) was evaluated by flow cytometry (n=3). **B** Statistical graph of transduction efficiency (EGFR+) (n=3). **C** Following the introduction of exogenous genes into TILs via lentiviral vectors, the proportion of TIGIT+ TILs was quantified by flow cytometry (n=3). **D** Statistical analysis chart of TIGIT+ TILs (n=3). **E** The secretion of IL-7 was quantified using the ELISA kit (n=3). **F** Western blotting was employed to assess the expression level of the exogenously introduced gene. **G** Statistical analysis of the viability of engineered TILs cultured in varying concentrations of IL-2 (n=3). **H** Statistical analysis of TIL yield (n=3). **I** Statistical analysis of the expansion fold of engineered TILs cultured in varying concentrations of IL-2 (n=3). **J** Statistical analysis chart of stem-like cells proportion within the CD8+ subset in engineered TILs (n=3). **K** Statistical analysis chart of stem-like cells proportion within the CD4+ subset in engineered TILs (n=3). **L** Statistical analysis chart of activation proportion of engineered TILs cocultured with cancer cells and dendritic cells (n=3). *p < 0.05, **p < 0.01, ***p < 0.001, and ****p < 0.0001.

**
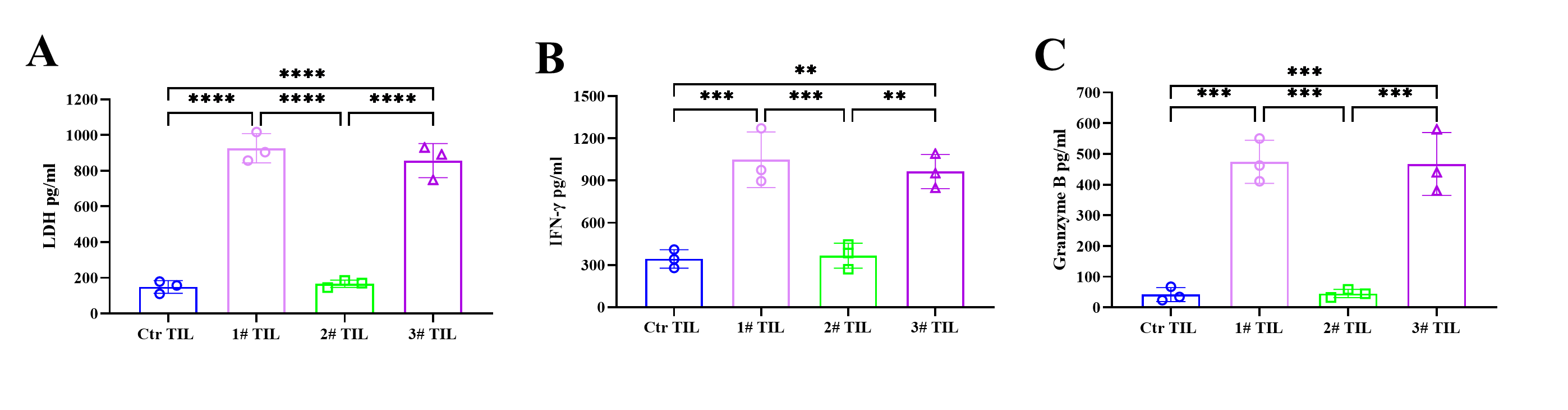
**

**Supplementary figure 2 Cytotoxicity analysis of engineered TILs**

**A** Following sustained challenge of the engineered TILs with tumor cells, the release amount of LDH was determined using the assay kit (n=3). **B** Following sustained challenge of the engineered TILs with tumor cells, the secretion of IFN-γ was quantified using the ELISA kit (n=3). **C** Following sustained challenge of the engineered TILs with tumor cells, the secretion of Granzyme B was quantified using the ELISA kit (n=3). *p < 0.05, **p < 0.01, ***p < 0.001, and ****p < 0.0001.

**
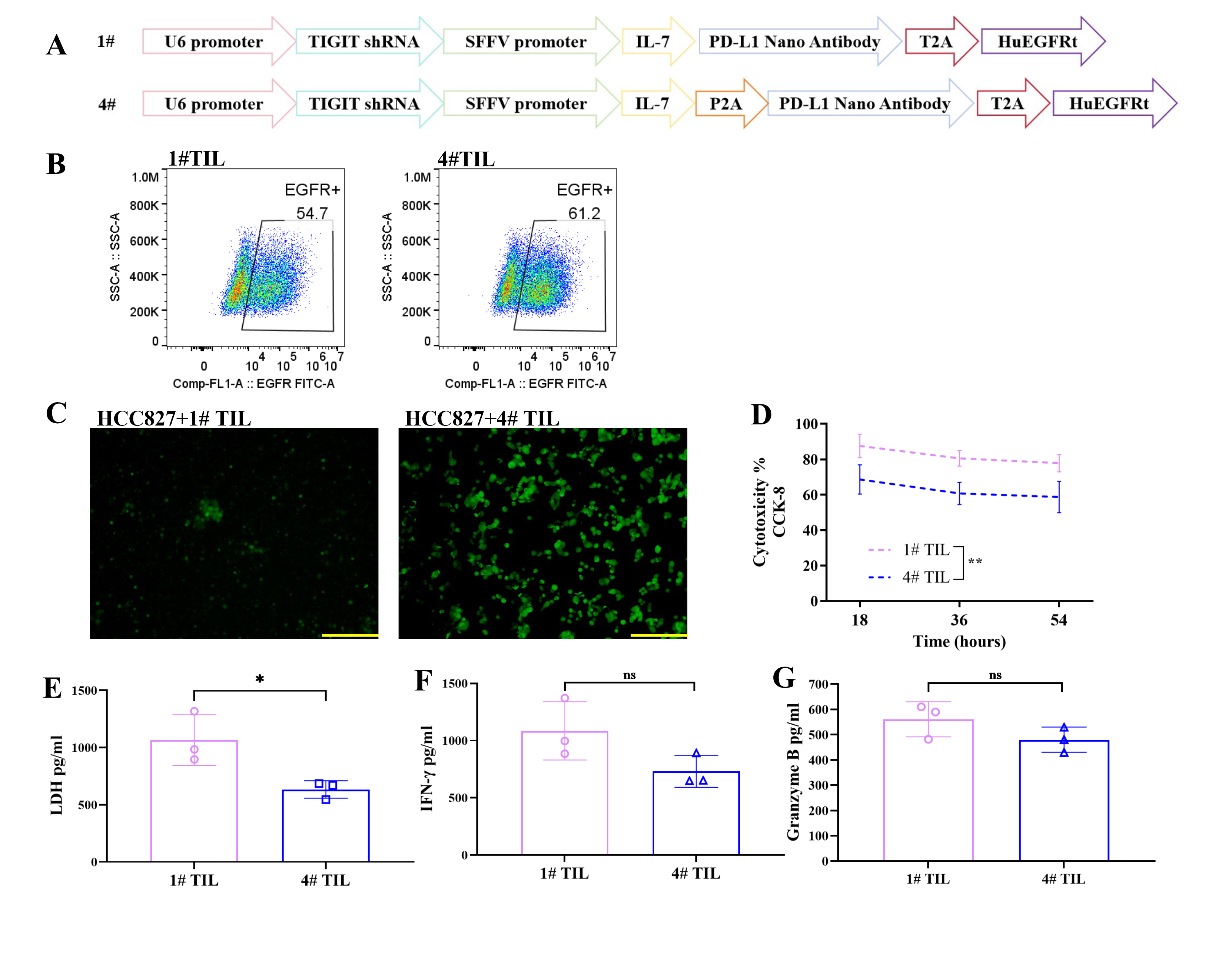
**

**Supplementary Figure 3 Cytotoxicity analysis of engineered TILs.**

**A** The Molecular framework of engineered TILs. **B** Following the introduction of exogenous genes into TILs via lentiviral vectors, the transduction efficiency (EGFR+) was evaluated by flow cytometry (n=3). **C** Following sustained challenge of the engineered TILs with tumor cells (GFP-labeled), the efficacy of tumor cell clearance was observed and photoed using fluorescence microscopy. bar=100μm (n=3). **D** Following sustained challenge of the engineered TILs with tumor cells, the efficacy of tumor cell clearance was quantified by CCK-8 assay (n=3). **E** Following sustained challenge of the engineered TILs with tumor cells, the release amount of LDH was determined using the assay kit (n=3). **F** Following sustained challenge of the engineered TILs with tumor cells, the secretion of IFN-γ was quantified using the ELISA kit (n=3). **G** Following sustained challenge of the engineered TILs with tumor cells, the secretion of Granzyme B was quantified using the ELISA kit (n=3). *p < 0.05, **p < 0.01, ***p < 0.001, and ****p < 0.0001.

**
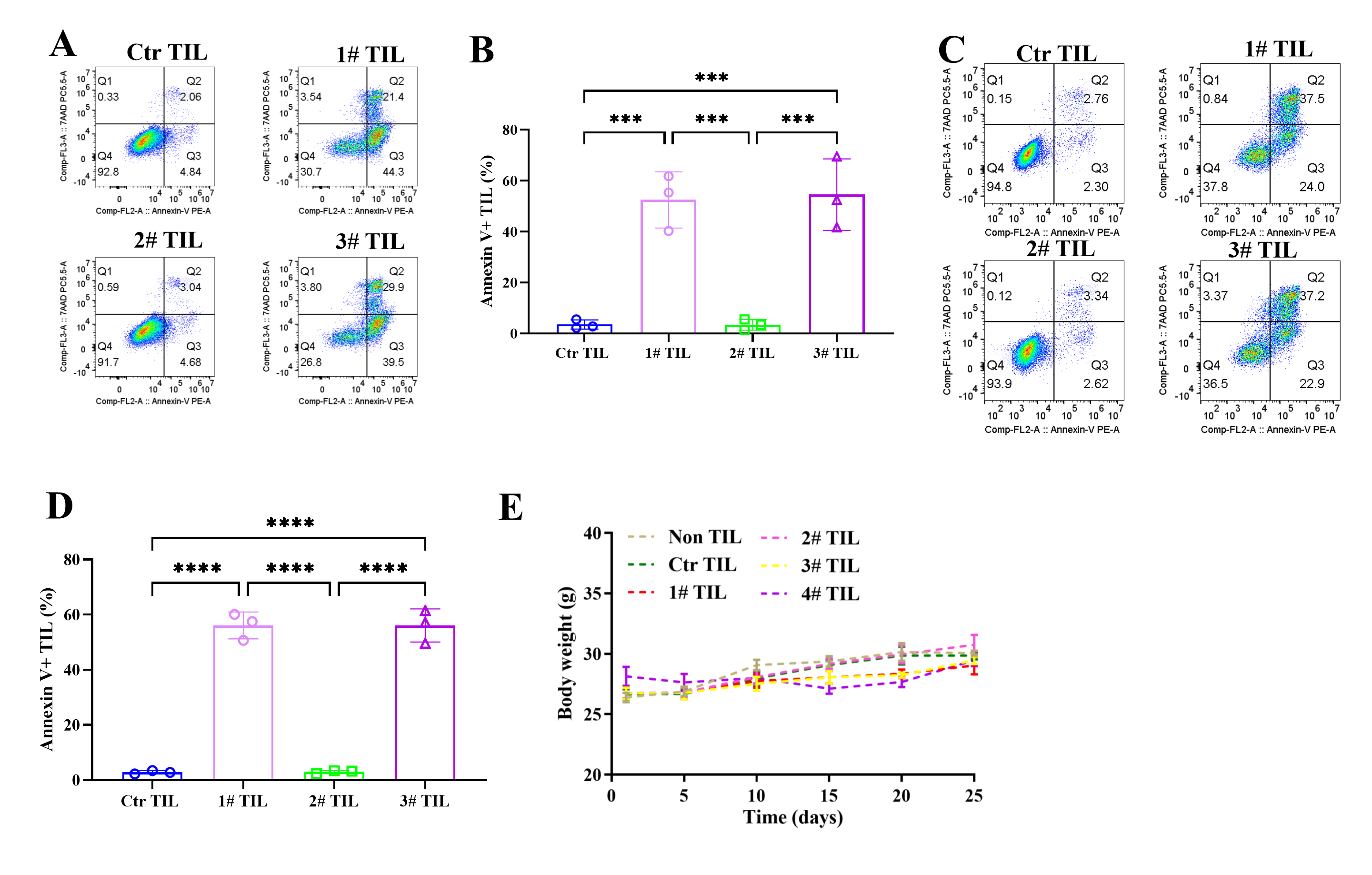
**

**Supplementary figure 4 Safety assessment of engineered TILs.**

**A** The apoptotic rate of engineered TILs induced by cetuximab through CDC were analyzed by flow cytometry (n=3). **B** Statistical analysis of engineered TILs apoptosis mediated by CDC (n=3). **C** The apoptotic rate of engineered TILs induced by cetuximab through ADCC were analyzed by flow cytometry (n=3). **D** Statistical analysis of engineered TILs apoptosis mediated by ADCC (n=3). **E** The changes in body weight of the mice throughout the text period (n=5). CDC, complement-dependent cytotoxicity; ADCC, antibody-dependent cellular cytotoxicity.

*p < 0.05, **p < 0.01, ***p < 0.001, and ****p < 0.0001.


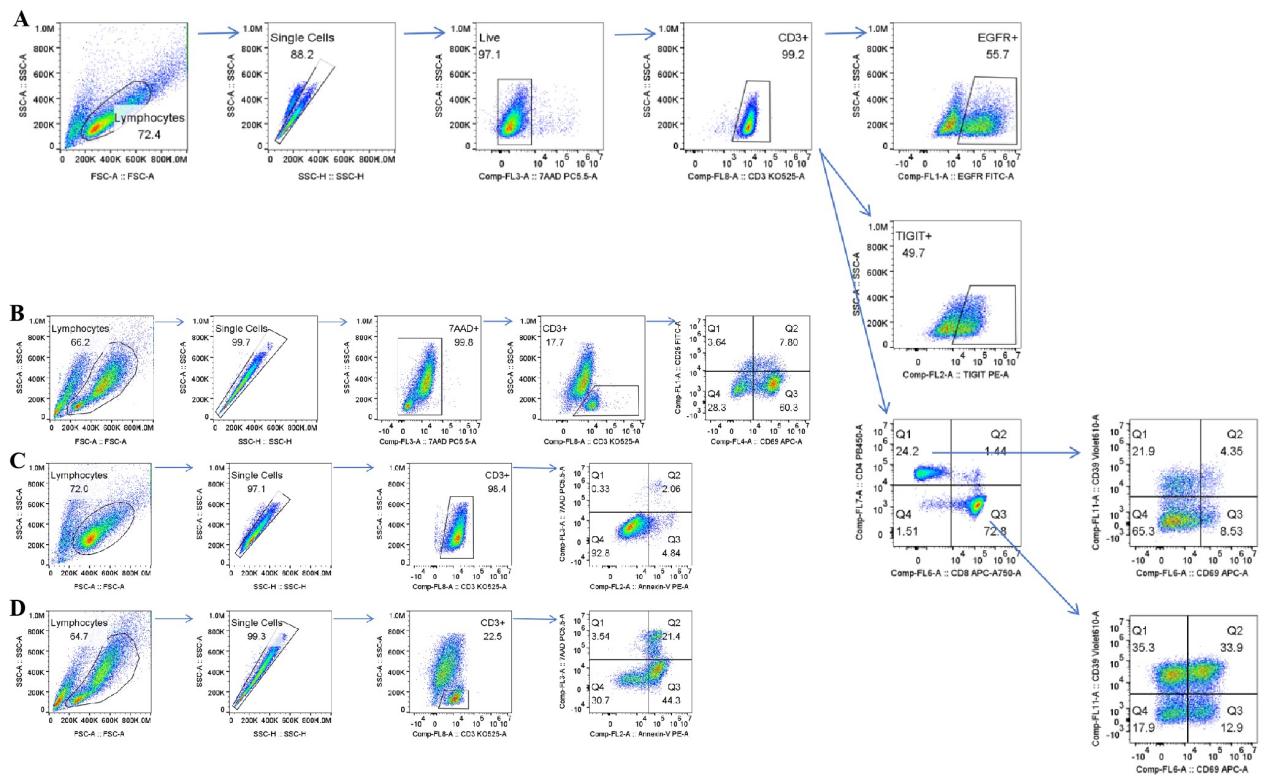


**Supplementary figure 5 Gating strategy of flow cytometry**

**A** Gating strategy EGFR+TILs, TIGIT+TILs,CD39-CD69-TILs. **B** Gating strategy CD25+CD69+TILs. **C** Gating strategy of apoptosis TILs induced by CDC. **D** Gating strategy apoptosis TILs induced by ADCC. CDC, complement-dependent cytotoxicity; ADCC, antibody-dependent cellular cytotoxicity.

| **Table1 Routine blood tests** | | | | | | | | | | | | | |
| --- | --- | --- | --- | --- | --- | --- | --- | --- | --- | --- | --- | --- | --- |
|  |  | WBC | Neu | Lym | Mon | Eos | Bas | Neu | Lym | Mon | Eos | Bas | RBC |
|  |  | 10^6/ml | 10^6/ml | 10^6/ml | 10^6/ml | 10^6/ml | 10^6/ml | % | % | % | % | % | 10^9/ml |
| Non TIL | Mean | 3.19 | 2.70 | 1.03 | 1.42 | 1.02 | 1.01 | 69.53 | 2.51 | 18.53 | 2.07 | 1.36 | 9.07 |
|  | SE | 0.16 | 0.17 | 0.01 | 0.05 | 0.02 | 0.01 | 2.14 | 0.36 | 2.14 | 0.18 | 0.09 | 0.09 |
| 1# TIL | Mean | 2.88 | 2.44 | 1.24 | 1.24 | 0.92 | 0.90 | 73.08 | 3.05 | 16.03 | 2.06 | 1.26 | 8.92 |
|  | SE | 0.13 | 0.14 | 0.01 | 0.04 | 0.01 | 0.02 | 2.05 | 0.62 | 1.60 | 0.36 | 0.09 | 0.05 |

| **Table2 Blood biochemical tests** | | | | | | | | | | | | | |  | | |
| --- | --- | --- | --- | --- | --- | --- | --- | --- | --- | --- | --- | --- | --- | --- | --- | --- |
|  |  | ALT | AST | ALP | TP | ALBIⅡ | CREA-S | UA | UREA | LDH | CK | Glu-G | TC | TG | LDL-C | HDL-C |
|  |  | U/L | U/L | U/L | g/L | g/L | μmol/L | μmol/L | mol/L | U/L | U/L | mmol/L | mmol/L | mmol/L | mmol/L | mmol/L |
| Non TIL | Mean | 50.36 | 134.68 | 103.24 | 46.52 | 33.80 | 26.28 | 105.48 | 13.34 | 482.36 | 646.20 | 14.45 | 10.87 | 10.14 | 9.56 | 10.62 |
|  | SE | 6.36 | 7.64 | 8.36 | 5.64 | 1.72 | 1.96 | 8.76 | 1.50 | 57.24 | 57.48 | 1.76 | 1.56 | 1.50 | 1.42 | 1.62 |
| 1# TIL | Mean | 56.76 | 148.60 | 106.76 | 51.16 | 34.68 | 28.36 | 106.84 | 13.25 | 540.36 | 744.60 | 14.28 | 10.74 | 9.95 | 9.59 | 10.67 |
|  | SE | 8.82 | 7.22 | 7.78 | 7.30 | 2.42 | 3.30 | 12.74 | 2.24 | 85.86 | 83.54 | 2.68 | 2.29 | 2.20 | 2.12 | 2.20 |
